# Exploring demographic and lifestyle associations with patient experience following telephone triage by a primary care doctor or nurse: secondary analyses from a cluster randomised controlled trial

**DOI:** 10.1136/bmjqs-2015-003937

**Published:** 2015-05-18

**Authors:** Fiona C Warren, Raff Calitri, Emily Fletcher, Anna Varley, Tim A Holt, Valerie Lattimer, David Richards, Suzanne Richards, Chris Salisbury, Rod S Taylor, John L Campbell

**Affiliations:** 1 Primary Care Research Group, University of Exeter Medical School, Exeter, UK; 2 School of Nursing Sciences, Faculty of Medicine and Health Sciences, University of East Anglia, Norwich, UK; 3 Department of Primary Care Health Sciences, University of Oxford, Oxford, UK; 4 Centre for Academic Primary Care, School of Social and Community Medicine, University of Bristol, Bristol, UK

**Keywords:** Primary care, General practice, Patient satisfaction

## Abstract

**Background:**

The ESTEEM trial was a cluster randomised controlled trial that compared two telephone triage management systems (general practitioner (GP) or a nurse supported by computer decision support software) with usual care, in response to a request for same-day consultation in general practice.

**Aim:**

To investigate associations between trial patients’ demographic, health, and lifestyle characteristics, and their reported experiences of care.

**Setting:**

Recruitment of 20 990 patients occurred between May 2011 and December 2012 in 42 GP practices in England (13 GP triage, 15 nurse triage, 14 usual care).

**Method:**

Patients reported their experiences via a postal questionnaire issued 4 weeks after their initial request for a same-day consultation. Overall satisfaction, ease of accessing medical help/advice, and convenience of care were analysed using linear hierarchical modelling.

**Results:**

Questionnaires were returned by 12 132 patients (58%). Older patients reported increased overall satisfaction compared with patients aged 25–59 years, but patients aged 16–24 years reported lower satisfaction. Compared with white patients, patients from ethnic minorities reported lower satisfaction in all three arms, although to a lesser degree in the GP triage arm. Patients from ethnic minorities reported higher satisfaction in the GP triage than in usual care, whereas white patients reported higher satisfaction with usual care. Patients unable to take time away from work or who could only do so with difficulty reported lower satisfaction across all three trial arms.

**Conclusions:**

Patient characteristics, such as age, ethnicity and ability to attend their practice during work hours, were associated with their experiences of care following a same-day consultation request in general practice. Telephone triage did not increase satisfaction among patients who were unable to attend their practice during working hours.

**Trial registration number:**

ISCRTN20687662.

## Introduction

Patient experience of care in general practice is routinely monitored in England using the national General Practice Patient Survey (GPPS).^1^ Recent analyses of GPPS data identified differential overall satisfaction with routine primary care across patient sociodemographic factors, such as age and ethnicity.^2^ Further analyses of GPPS data have indicated that interpersonal aspects of care were the strongest drivers of patient satisfaction, whereas ease of access to care was a weaker driver of patient satisfaction.^3^ However, younger adult patients (aged 18–25 years) valued access relatively more highly (compared with patients aged 55–64 years); patients from Asian and Chinese ethnic backgrounds valued telephone access relatively more highly (compared with white patients). At present, little is known about how the management of appointment requests, for example through the use of triage systems, may have an impact on patient experience of care. While providing important data on patients’ experiences in primary care, both routine and out of hours, GPPS does not specifically address patient experience following requests for a prompt consultation within routine practice hours, for example a telephone request to see a general practitioner (GP) that same day.

A systematic review of patient satisfaction with telephone triage models^4^ identified two randomised controlled trials (RCTs).^5^ ^6^ The more recent of these was conducted in two practices in Scotland and randomised patients who contacted their practice to request a same-day appointment to either a face-to-face appointment on that day or to telephone triage by a GP.^6^ That trial found no significant difference in patient satisfaction; however, the control group received a same-day face-to-face GP consultation, which may not routinely be available to all patients requesting such a consultation in all practices. Thus, the comparison of GP telephone triage versus a same-day face-to-face GP consultation may not be generalisable to many practices.

The ESTEEM three-arm cluster RCT investigated the effects of two forms of triage, one led by GPs and the other led by nurses using computer-supported decision-making software, compared with usual care (defined as the care that the patient would normally receive from his/her practice when requesting a same-day GP consultation). The design and primary findings of ESTEEM have been described elsewhere,^7–9^ as well as a detailed account of the process evaluation^10^ and a discussion of differences in communication comparing GPs and nurses performing telephone triage.^11^ Conducted in four regions of England, this large cluster RCT (randomisation was conducted at the level of the practice) collected data from 20 990 patients within 42 GP practices. The aim of the trial was to compare the effects of the two triage systems with usual care, with regard to healthcare resource use, safety, health-related outcomes and patient satisfaction outcomes, among patients who had requested a same-day face-to-face consultation with a GP. Initial analyses of the trial data indicated that patients’ reports of their experience of care were generally positive, although there was some evidence that nurse triage was less positively regarded by patients compared with both usual care and GP triage.^8^ ^9^

This paper presents more detailed analyses of the ESTEEM trial data regarding patients’ experience of, or satisfaction with, care. The secondary analyses reported here sought to (i) identify any patient characteristics associated with patient experience and (ii) determine whether there were any differences in reporting of experience across trial arms among different groups as defined by patient characteristics. We address these issues with regard to patient sociodemographic characteristics (gender, age, ethnic group and deprivation status) and health/lifestyle characteristics (presence of a long-standing health condition and self-reported ability to take time away from work during the patient's typical working hours if relevant).

## Methods

The data collection and analysis methods of the ESTEEM trial have been reported elsewhere.^7–9^

### Patient questionnaire

All eligible patients within ESTEEM were sent a postal questionnaire (including a participant information sheet and reply-paid envelope) 4 weeks after the initial same-day consultation request. Further details on the development of the ESTEEM questionnaire have been reported elsewhere.^9^ To maximise response rates, non-respondents to initial mailings were sent up to two reminders (after 2 and 4 weeks had elapsed respectively). Implied consent to participate was evidenced by return of the questionnaire. The questionnaire included six evaluative items inviting the patient (or a parent/carer) to rate aspects of the care received by the patient on the day of the consultation request. We investigated three aspects of patient care: (i) overall satisfaction with care (scored on a Likert scale of 1–5: 1—very satisfied; 2—fairly satisfied; 3—neither satisfied not dissatisfied; 4—fairly dissatisfied; 5—very dissatisfied); (ii) ease of getting medical help or advice for the problem (scored on a Likert scale of 1–5: 1—very easy; 2—fairly easy; 3—neither easy nor difficult; 4—fairly difficult; 5—very difficult); and (iii) convenience of care (scored on a Likert scale of 1–4: 1—very convenient; 2—fairly convenient; 3—not very convenient; 4—not at all convenient).

### Patient characteristics

Data on age and gender were available for all patients within the trial (supplied from practice records), with data on deprivation being available for patients whose postcode (supplied from practice records) could be mapped to an Indices of Multiple Deprivation (IMD) 2010 score.^12^ Data on ethnicity, presence of a long-standing health condition and ease of taking time away from work were only available if the patient returned a questionnaire with the relevant item completed. Age was categorised into six ranges: 0–4 years; 5–11 years; 16–24 years; 25–59 years (reference category); 60–74 years; 75 years and older (patients aged 12–15 years were excluded from the trial due to reasons of confidentiality, as some patients in this age group may wish their parents/guardians to be unaware of their clinician consultation; should the parents/guardians open a questionnaire addressed to the patient, they would be alerted to this fact).

The ESTEEM questionnaire invited patients to report their ethnic group using five categories. Ethnic group was then dichotomised for the purposes of these analyses as ‘white’ and ‘other ethnic group’ (comprising Mixed/multiple ethnic groups, Asian/Asian British, Black/African/Caribbean/Black British, and Other ethnic group) due to the small number of patients from ethnic minority backgrounds. Deprivation status was based on IMD 2010 scores mapped to the patient's residential postcode. Deprivation was divided into five quintiles based on rank (using national quintiles as cut-off points), using the least deprived quintile as the reference category. Patients reported how easy it was for them to attend a GP consultation at their practice during working hours (categorised as easily, with difficulty or unable to attend during working hours), with the option to record if the question was not relevant (eg, if the patient was a child or non-working adult); the ‘not relevant’ category was used as the reference group within analyses. Patients also reported the presence/absence of a long-standing health condition.

### Statistical methods

The satisfaction/experience outcome variables were linearised on a scale of 0–100 (lower values indicating lower levels of negative response) to facilitate ease of interpretation.^2^ On this scale, a difference of <3 points is considered to be of small magnitude in practical terms.^13^ All analyses took the form of multilevel linear regression models (using the Stata command ‘xtmixed’) with a random effect on practice (cluster).^14^ The multilevel structure provides a ‘within practice’ approach, evaluating differences across sociodemographic characteristics within each practice and allowing each practice to have its own baseline score within the model. This approach allows for ‘clustering’ of participants with specific sociodemographic characteristics within a practice.^2^ However, our aim was not to evaluate the degree of variation in an outcome that was attributable to practice level variation,^14^ as this was not an issue of interest in our analyses; rather, we aimed to evaluate the effects of sociodemographic covariates after adjusting for practice level variables and accounting for the multilevel nature of the data. All minimisation variables used in the cluster randomisation procedure of the ESTEEM trial (practice list size (small, medium, large), practice deprivation (deprived/non-deprived) and location (Bristol, Devon, Norwich, Warwick)^7–9^) were included as fixed effect variables in all analyses as was trial arm (using usual care as the reference group).

A series of multivariable models were fitted to investigate potential associations between the sociodemographic/lifestyle variables and each outcome variable individually. Potential interactions of sociodemographic variables with trial arm were also investigated, by inclusion of one potential interaction term within an individual model. Although the mode of management actually received by individual patients varied within each arm as well as across arms,^9^ we have taken the pragmatic approach of analysing patient experience outcomes by allocated trial arm rather than by the mode of management received. Sociodemographic/lifestyle variables that were found to be not significantly associated with an individual outcome variable (within a multivariable model) were excluded from the final model for that outcome variable. The p value for statistical significance was set at <0.05 for main effects and <0.1 for an interaction term.

Marginal means were reported for statistically significant interaction effects between trial arm and the specified sociodemographic/lifestyle factor. Marginal mean scores indicated the expected score for specified patient characteristic groups (eg, by gender) across the three trial arms individually (eg, male patients receiving usual care, male patients receiving GP triage, and so on to encompass the six possible combinations of three trial arms and two genders). Such scores are based on the assumption that all patients in the model take the specified values for trial arm and gender, while all other variables within the model retain their observed values.

All analyses were performed on an intention-to-treat basis (not all patients received the management method allocated to their practice^9^) using complete case data. All analyses were conducted using Stata v.12.

## Results

### Study population characteristics

Of the 20 990 patients within ESTEEM, 12 132 (58%) returned a questionnaire that included at least one completed question. A smaller proportion of patients who were sent a questionnaire responded to the question on overall satisfaction in nurse triage (3704/7012; 53%) compared with 4093/7283 (56%) in usual care and 4034/6695 (60%) in GP triage. Of 12 132 questionnaire respondents, 11 831 (98%) completed the question regarding overall satisfaction; their demographic characteristics are presented in table 1; demographic data for questionnaire respondents and a detailed analysis of the factors associated with questionnaire response are reported elsewhere.^9^ The predominantly female sample reflected the overall study sample. However, patients in older age groups (60 and over) were over-represented within the sample of patients who provided satisfaction data compared with the overall study sample, whereas young adults (aged 16–24 years) were under-represented. Patients in the most deprived quintile were under-represented both in the overall study sample and the sample providing experience data.^9^ Of 12 132 questionnaire respondents, 11 119 (92%) provided data on the ease of getting medical help or advice; 11 783 (97%) provided data on convenience of care. The frequencies for each response category are set out in the ESTEEM report.^9^

**Table 1:** Baseline patient sociodemographic characteristics for patients who responded to overall satisfaction question

	Usual care (UC; N=4093)	GP triage (GPT; N=4034)	Nurse triage (NT; N=3704)
Individual patient characteristics derived from practice data*†			
Gender; n (%)			
Male	1579 (38.6)	1601 (39.7)	1417 (38.3)
Female	2514 (61.4)	2433 (60.3)	2287 (61.7)
Age (years); mean (SD)	46.8 (24.0)	49.6 (24.8)	47.3 (25.4)
By category; n (%)			
Under 5	347 (8.5)	325 (8.1)	380 (10.2)
5–11	222 (5.4)	210 (5.2)	214 (5.8)
16–24	297 (7.3)	227 (5.6)	220 (5.9)
25–59	1757 (42.9)	1591 (39.4)	1487 (40.2)
60–74	1047 (25.6)	1052 (26.1)	899 (24.3)
75 and over	423 (10.3)	629 (15.6)	504 (13.6)
Deprivation (IMD 2010† score); mean (SD), n	16.6 (9.6), 4069	16.2 (10.6), 4025	16.2 (10.5), 3673
Deprivation (IMD 2010† quintile based on rank); n (%)			
Quintile 1 (least deprived)	817 (20.1)	779 (19.4)	870 (23.7)
Quintile 2	1136 (27.9)	1220 (30.3)	1038 (28.3)
Quintile 3	1089 (26.8)	1198 (29.8)	864 (23.5)
Quintile 4	824 (20.3)	590 (14.7)	665 (18.1)
Quintile 5 (most deprived)	203 (5.0)	238 (5.9)	236 (6.4)
Individual patient characteristics derived from questionnaire			
Ethnicity—by ethnic group; n (%)			
White	3927 (96.5)	3851 (96.1)	3498 (95.2)
Other ethnic group	143 (3.5)	158 (3.9)	175 (4.8)
Total N	4070	4009	3673
Able to attend surgery during work hours; n (%)			
Not relevant	1956 (48.6)	2049 (51.8)	1811 (49.8)
Yes, easily	792 (19.7)	787 (19.9)	721 (19.8)
Yes, with difficulty	877 (21.8)	829 (20.9)	768 (21.1)
No	400 (9.9)	294 (7.4)	335 (9.2)
Total N	4025	3959	3635
Long-standing health conditions; n (%)			
Yes	1923 (48.0)	1973 (50.1)	1669 (46.0)
No	2087 (52.0)	1967 (49.9)	1957 (54.0)
Total N	4010	3940	3626

*Age and gender derived directly from practice records.

†IMD 2010 score and rank derived from residential postcode data (provided by the patient's practice) mapped to lower super output area; https://www.gov.uk/government/publications/english-indices-of-deprivation-2010.

IMD, Indices of Multiple Deprivation.

### Overall satisfaction with care

Overall, patients were satisfied with their care, with approximately 90% of responding patients in each trial arm reporting that they were ‘very’ or ‘fairly’ satisfied with their care, although only 59% of responding patients in the nurse triage arm were ‘very’ satisfied, compared with around 65% in the usual care and GP triage arms.^9^ Gender and patient deprivation were not significantly associated with overall satisfaction and were therefore excluded from subsequent analyses. Older patients were more satisfied compared with adults aged 25–59 years; for example, patients aged 75 years and over had a mean difference in score of −3.83, with a 95% CI of −5.25 to −2.40 (table 2, Model A), whereas young adults (aged 16–24 years) were less satisfied (mean difference 4.35, 95% CI 2.74 to 5.97; table 2, Model A). Patients from ethnic minorities reported reduced satisfaction compared with white patients (mean difference 5.00, 95% CI 2.96 to 7.04; table 2, Model A). Also, patients who reported that they were unable to attend the practice during working hours, or could only do so with difficulty, were less satisfied than those patients for whom this issue was not relevant (mean difference (95% CI) 5.41 (3.89 to 6.94) and 2.46 (1.29 to 3.62) respectively; table 2, Model A); there was little evidence to indicate that this reduced satisfaction varied by trial arm (see online supplementary appendix table A1). Patients who were easily able to attend the practice during working hours did not report significantly lower satisfaction than those patients for whom attendance during working hours was not relevant. The presence of a long-standing health condition was also significantly associated with reduced satisfaction, but to a small extent. Patients from ethnic minority backgrounds and patients who were unable to take time away from work to attend their surgery during working hours both reported poorer satisfaction of approximately 5 points compared with the relevant reference group.

**Table 2:** Overall satisfaction with care: sociodemographic associations and interactions with trial arm

	Mean difference in overall satisfaction with care* (95% CI)	Global p value	
*Model A†: N=11 343*			
Trial arm			
Reference; usual care			
GP triage	1.18 (−0.69 to 3.06)	<0.001	
Nurse triage	3.78 (1.88 to 5.69)		
Patient characteristic			
Age (reference: 25–59 years)			
Under 5 years	0.90 (−0.62 to 2.42)	<0.001	
5–11 years	0.60 (−1.21 to 2.41)		
16–24 years	4.35 (2.74 to 5.97)		
60–74 years	−2.70 (−3.81 to −1.60)		
75 years and over	−3.83 (−5.25 to −2.40)		
Ethnic group (reference; white)			
Other ethnic group	5.00 (2.96 to 7.04)	<0.001	
Ease of taking time away from work to attend surgery (reference: not relevant‡)			
Can take time away from work easily	−0.63 (−1.75 to 0.49)	<0.001	
Can take time away from work with difficulty	2.46 (1.29 to 3.62)		
Cannot take time away from work	5.41 (3.89 to 6.94)		
Presence of long-standing health condition (reference: none)			
Long-standing health condition present	1.83 (0.98 to 2.67)	<0.001	

	Mean difference in overall satisfaction with care* (95% CI)	Global p value	Marginal mean value§ (95% CI)
*Model B¶: N=11 343*			
GP triage interaction with ethnicity			
Other ethnic group	−5.21 (−10.17 to −0.25)	0.101	
Nurse triage interaction with ethnicity			
Other ethnic group	−1.44 (−6.41 to 3.52)		
Marginal mean values			
Usual care			
White			11.0 (9.7 to 12.3)
Other ethnic group			18.3 (14.6 to 22.0)
GP triage			
White			12.4 (11.1 to 13.7)
Other ethnic group			14.5 (11.0 to 18.1)
Nurse triage			
White			14.8 (13.5 to 16.2)
Other ethnic group			20.7 (17.2 to 24.2)

*Positive mean difference indicates reduced overall satisfaction with care in comparator group; scale 0–100.

†Adjusted for practice site, size and practice-level deprivation.

‡Patient does not work, for example, a child or non-working adult.

§Higher value indicates reduced satisfaction; scale 0–100.

¶Adjusted as for Model A, with inclusion of interaction between trial arm and ethnic group.

GP, general practitioner.

**Figure 1:**
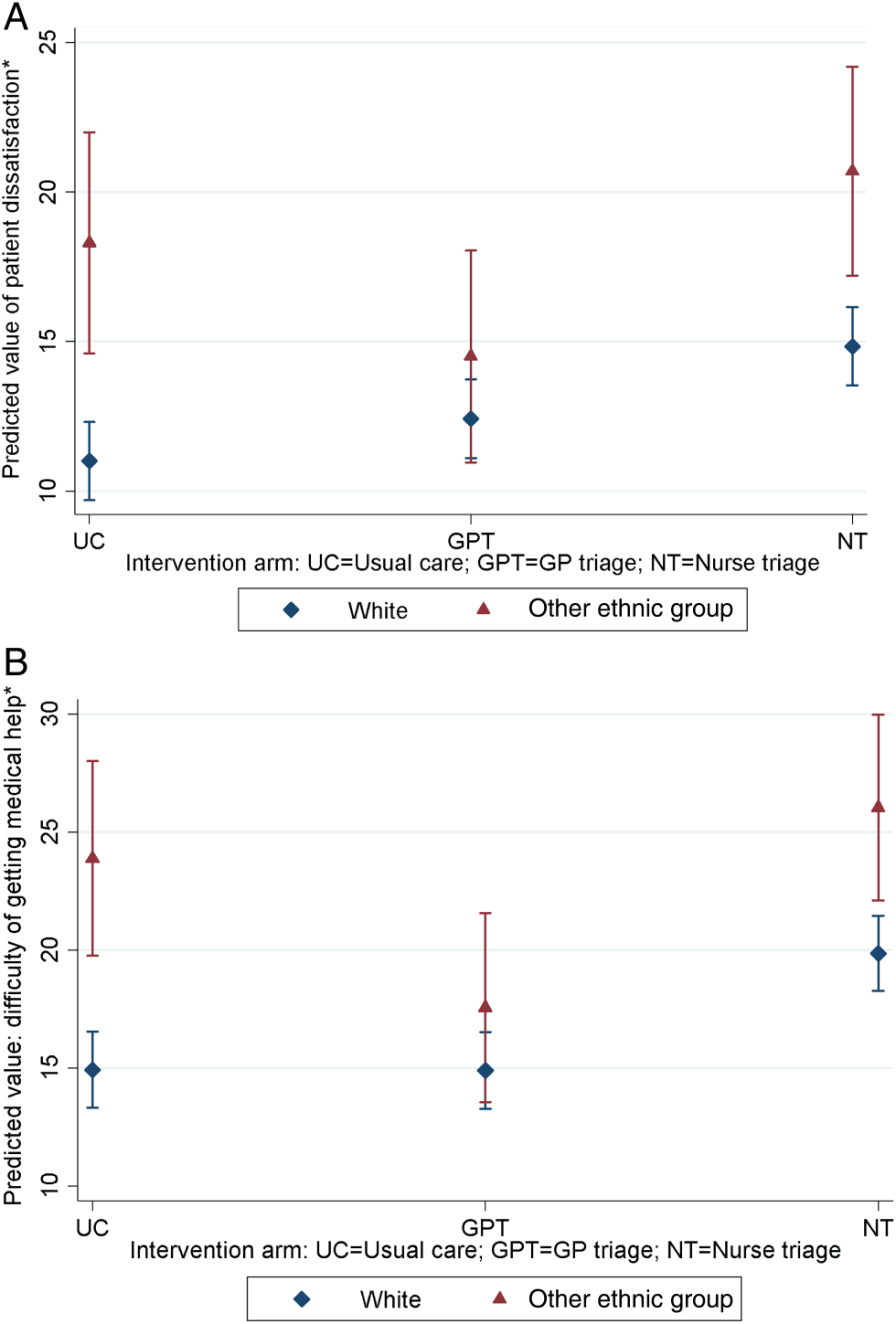
Marginal mean values (with 95% CI) across ethnic groups for (A) overall satisfaction with care and (B) ease of getting medical help or advice. *Lower values indicate higher overall satisfaction with care/greater ease of getting medical help or advice.

While there was little evidence for a significant interaction between GP triage and nurse triage compared with usual care and individual sociodemographic characteristics, there was some evidence to indicate an interaction between ethnic group and GP triage only compared with usual care (p value 0.040). Patients from ethnic minorities reported lower satisfaction compared with white patients for all three trial arms, whereas for GP triage, the marginal mean score was closer to that of white patients (table 2, Model B; figure 1A). This interaction between ethnicity and trial arm indicates a different pattern in satisfaction across ethnic groups, whereby white patients reported greatest satisfaction with usual care, followed by GP triage and then nurse triage. In contrast, the pattern seen among patients from ethnic minorities indicated that GP triage was associated with greatest satisfaction, followed by usual care and then nurse triage. The full results of Model A are presented in online supplementary appendix table A1. No other statistically significant interactions were observed between trial arm and sociodemographic or lifestyle factors included in Model A (see online supplementary appendix table A1, Models H–J) or between non-significant sociodemographic characteristics (gender and patient deprivation) when individually added to Model A with the appropriate interaction term (data not presented).

### Ease of getting medical help/advice

Gender and patient deprivation were not significantly associated with this aspect of patient care, and were therefore excluded from subsequent analyses. Patients in the nurse triage arm reported increased difficulty in obtaining medical help/advice compared with usual care (mean difference 4.81, 95% CI 2.49 to 7.12; table 3, Model C). There was some evidence for an interaction between trial arm and ethnicity (p value 0.079). Patients from ethnic minorities reported relatively greater ease of getting medical help/advice in the GP triage arm (versus usual care) compared with white patients (p value 0.025), although again, patients from ethnic minorities appeared to report poorer absolute scores than white patients across all three trial arms (eg, in the nurse triage arm, the marginal mean score for white patients was 19.9, 95% CI 18.3 to 21.5, compared with 26.0, 95% CI 22.1 to 30.0 for patients from ethnic minorities; table 3, Model D; figure 1B). The full results of Model C are presented in online supplementary appendix table A2. No significant interactions between other patient characteristics included in Model C were observed (see online supplementary appendix table A2, Models K–M); nor were there any significant interactions between trial arm and gender or deprivation when individually added to Model C with the appropriate interaction term (data not presented).

**Table 3:** Ease of getting medical help or advice: sociodemographic associations and interactions with trial arm

	Mean difference in ease of getting medical help or advice* (95% CI)	Global p value	
*Model C†: N=10 683*			
Trial arm			
Reference; usual care			
GP triage	−0.30 (−2.59 to 1.98)	<0.001	
Nurse triage	4.81 (2.49 to 7.12)		
Patient characteristic			
Age (reference: 25–59 years)			
Under 5 years	1.19 (−0.47 to 2.86)	<0.001	
5–11 years	1.54 (−0.44 to 3.51)		
16–24 years	2.59 (0.83 to 4.35)		
60–74 years	−3.22 (−4.44 to −1.99)		
75 years and over	−3.36 (−4.96 to −1.76)		
Ethnic group (reference; white)			
Other ethnic group	5.86 (3.60 to 8.12)	<0.001	
Ease of taking time away from work to attend surgery (reference: not relevant‡)			
Can take time away from work easily	−0.87 (−2.11 to 0.38)		
Can take time away from work with difficulty	3.74 (2.46 to 5.03)	<0.001	
Cannot take time away from work	7.65 (5.97 to 9.32)		
Presence of long-standing health condition (reference: no long-standing health condition)			
Long-standing health condition present	2.01 (1.07 to 2.94)	<0.001	

	Mean difference in overall satisfaction with care* (95% CI)	Global p value	Marginal mean value§ (95% CI)
*Model D¶: N=10 683*			
GP triage interaction with ethnicity			
Other ethnic group	−6.29 (−11.78 to −0.79)		
Nurse triage interaction with ethnicity		0.079	
Other ethnic group	−2.78 (−8.28 to 2.73)		
Marginal mean values			
Usual care			
White			14.9 (13.3 to 16.5)
Other ethnic group			23.9 (19.7 to 28.0)
GP triage			
White			14.9 (13.3 to 16.5)
Other ethnic group			17.6 (13.6 to 21.6)
Nurse triage			
White			19.9 (18.3 to 21.5)
Other ethnic group			26.0 (22.1 to 30.0)

*Positive mean difference indicates increased difficulty in getting medical help or advice in comparator group; scale 0–100.

†Adjusted for practice site, size and practice-level deprivation.

‡Patient does not work, for example, a child or non-working adult.

§Higher value indicates increased difficulty in getting medical help or advice; scale 0–100.

¶Adjusted as for Model C, with inclusion of interaction between trial arm and ethnic group.

GP, general practitioner.

### Convenience of care

Convenience of care demonstrated slightly different patterns of association with patient characteristics. Although little evidence was found to support an association between gender and convenience of care, strong evidence was found to support an association between patient deprivation and convenience of care (p value 0.001). This association appeared to be driven by increased convenience reported by patients in the most deprived quintile compared with patients in the least deprived quintile (mean difference −4.05, 95% CI −6.35 to −1.75; table 4, Model E). There was some evidence for an interaction effect between deprivation and trial arm (p value 0.041). This interaction appeared to be driven by lower reported convenience for nurse triage by patients in the less deprived groups (based on marginal mean values; eg, in the usual care arm the marginal mean for patients in the least deprived quintile was 12.8 (95% CI 10.4 to 15.1), in the GP triage arm 14.0 (95% CI 11.5 to 16.6), and in the nurse triage arm 21.3 (95% CI 19.0 to 23.7); supplementary online appendix table A3, Model G), although the global p value (0.130) for the interaction between nurse triage compared with usual care, and deprivation group, was not significant. Some evidence for an interaction between age and trial arm was observed (p value 0.072); the interaction between age and nurse triage only versus usual care had a p value of 0.019. This interaction appeared to be driven by relatively lower convenience reported by patients (or more accurately their parents/guardians) in the 5–11 years age bracket compared with patients aged 25–59 years in the nurse triage arm (marginal mean for patients aged 5–11 years 26.7, 95% CI 23.3 to 30.2, for patients aged 25–59 years 18.8, 95% CI 16.9 to 20.8; online supplementary appendix table A3, Model F). The results of Model E are presented in full in online supplementary appendix table A4. No further significant interactions were found between trial arm and sociodemographic and lifestyle factors included in Model E (online supplementary appendix table A4, Models O–Q) or between trial arm and gender when added to Model E with the appropriate interaction term (data not presented).

**Table 4:** Convenience of care: sociodemographic associations and interactions with trial arm

	Mean difference in convenience of care* (95% CI)	Global p value
*Model E†: N=11 243*		
Trial arm		
Reference: usual care		
GP triage	1.99 (−0.49 to 4.47)	<0.001
Nurse triage	5.69 (3.19 to 8.20)	
Patient characteristic		
Age (reference: 25–59 years)		
Under 5 years	3.11 (1.48 to 4.74)	
5–11 years	4.28 (2.35 to 6.21)	
16–24 years	4.24 (2.51 to 5.97)	<0.001
60–74 years	−3.35 (−4.54 to −2.17)	
75 years and over	−2.68 (−4.21 to −1.15)	
Patient deprivation‡ (reference: Quintile 1; least deprived)		
Quintile 2	−1.27 (−2.54 to <−0.01)	
Quintile 3	−1.05 (−2.39 to −0.28)	0.001
Quintile 4	−0.01 (−1.59 to 1.57)	
Quintile 5	−4.05 (−6.35 to −1.75)	
Ethnic group (reference: white)		
Other ethnic group	6.36 (4.16 to 8.56)	<0.001
Ease of taking time away from work to attend surgery (reference: not relevant§)		
Can take time away from work easily	−1.56 (−2.76 to −0.36)	
Can take time away from work with difficulty	4.64 (3.39 to 5.89)	<0.001
Cannot take time away from work	9.02 (7.39 to 10.65)	
Presence of long-standing health condition (reference: no long-standing health condition)		
Long-standing health condition present	1.90 (1.00 to 2.81)	<0.001

*Positive mean difference indicates lower convenience of care in comparator group; scale 0–100.

†Adjusted for practice site, size and practice-level deprivation.

‡IMD 2010 score and rank derived from residential postcode data mapped to lower super output area; https://www.gov.uk/government/publications/english-indices-of-deprivation-2010.

§Patient does not work, for example, a child or non-working adult.

GP, general practitioner; IMD, Indices of Multiple Deprivation.

## Discussion

### Summary

Based on secondary analyses of a cluster RCT (ESTEEM) that investigated the clinical effectiveness of two telephone triage management systems for patients requesting a same-day consultation with a GP,^8^ ^9^ we sought to explore whether patients’ reported experiences of care were associated with their sociodemographic, health, and lifestyle characteristics.

We found little evidence for a significant interaction with regard to overall satisfaction between all three trial arms and sociodemographic groups. However, there was a significant interaction between GP triage only versus usual care and ethnic group with regard to overall patient satisfaction, and between trial arms and ethnic group regarding ease/difficulty of getting medical help or advice. Patients from ethnic minorities, although reporting less positively than white patients on both of these outcomes, reported scores closer to those of white patients when receiving GP triage than when receiving either usual care or nurse triage. A possible explanation for this could be that patients from ethnic minority backgrounds value rapid access to a GP more highly as a driver of satisfaction compared with white patients, and that telephone access is acceptable. However, a similar effect was not found with nurse triage, possibly indicating that rapid telephone access to a nurse is less acceptable than equivalent access to a GP.

We observed reduced convenience reported by parents/guardians of children aged 5–11 years in the nurse triage arm, which may be due to the inconvenience of waiting for a call back from the nurse at a time when the child is attending school. Patients in the nurse triage arm were also more likely (compared with patients receiving GP triage) to receive triage and then to be called in to the practice for a face-to-face consultation with a GP or nurse.^9^ This may be a further source of inconvenience when the child is attending school and the parents/guardians may be at work. There is some evidence to indicate that delays in receiving care and/or uncertainty of what to do while waiting for definitive care may also be a driver of dissatisfaction,^15^ and these issues may be heightened for parents of children in this age range, and are less well addressed by nurse triage than other management approaches.

These analyses indicate that triage systems do not alleviate the difficulties working patients may have in obtaining care in a flexible manner. It is possible that the telephone call back approach employed in both triage arms may have been inconvenient for working patients, due to difficulties in receiving calls while travelling to or being at work, for example due to being unable to stop their work to take the call or due to privacy issues in the workplace. The telephone triage took place during regular surgery hours, so this may explain why these outcomes (satisfaction, convenience, and ease of obtaining medical help or advice) did not appear to be more positively reported for patients with difficulty attending their surgery during regular hours in the triage arms.

### Strengths and limitations

ESTEEM was a large multicentre trial, which examined a range of aspects of patient experience. The large size of the trial and availability of data for many patients on a range of health and lifestyle factors as well as sociodemographic factors enabled us to undertake these analyses. Our response rate of 58% is relatively high for this type of patient survey within a general practice setting, for example, by comparison with the annual iterations of GPPS.^1^ A survey in the Netherlands suggested that non-response bias was small with regard to overall satisfaction with out-of-hours primary care.^16^ Therefore, we felt that our sample was sufficiently large to militate against the use of data imputation methods. However, the ESTEEM trial was not powered to detect interactions with regard to patient satisfaction, and therefore the results of these exploratory analyses should be viewed with caution.

Practices were given a 3-week ‘run-in’ period to establish the new routine of the triage system,^9^ but we acknowledge that this is a short time for staff and patients to become accustomed to the new approach. It is possible that reduced satisfaction in the triage arms may be due to this period of transition and that patient experience scores may improve as the triage system becomes more familiar to both staff and patients.

For safety reasons, patients were excluded from the trial if they were unable to communicate effectively by telephone, for example due to hearing/speech difficulties or difficulties with spoken English. In view of this, the reduced satisfaction reported by patients from ethnic minority backgrounds may be less likely (compared with other reports of reduced satisfaction among patients from ethnic minorities^2^ ^17^ ^18^) to have arisen due to difficulties with communication in spoken English (although we acknowledge that verbal communication in a second language, even if of a high standard, may be more difficult than communicating in the native language, especially when using the telephone, as visual cues are not available). However, a limitation of these analyses was that due to the small number of patients from minority ethnic groups, it was necessary to dichotomise ethnic group (into ‘white’ and ‘other ethnic group’ categories); hence, we were unable to investigate differences in experience among patients from different ethnic minority groups. The exclusion of patients who were unable to communicate effectively in English using the telephone may have contributed to the small number of patients from ethnic minority backgrounds. Furthermore, we did not elicit information on English language ability, which may have been associated with patient experience.

A further limitation was that we did not collect data regarding the patient's presenting complaint—the reason for the patient's initial request for a same-day GP consultation. Hence, we were unable to link satisfaction to the nature and/or severity of the medical problem resulting in the initial same-day consultation request. Such data may have been of particular relevance with regard to patients with a long-standing health condition (although we did not collect any information regarding the nature and/or severity of long-standing health conditions), older patients or children, in terms of trying to understand reasons for reporting their experience of care as they did. Nor did we request patients who were unable to take time away from work (or were able to do so only with difficulty) to report their occupation or typical working hours; such information may have provided insights into the reduced satisfaction among these patients. We did not aim to link patient experience with the timeliness of care or the quality of clinical care received; therefore, we are unable to comment on the potential association between patients’ reported experience of care and objective measures of the quality of care.

Participants were requested to report their experience of care received approximately 4 weeks previously. Although this is a relatively short time period, there is a possibility for recall bias, with regard to identifying the specific date in question, and with regard to recalling experience of care, especially if the patient is a frequent attender at his/her practice. For example, older patients may not accurately report health service resource use over a relatively short time frame (less than 12 weeks).^19^ Such difficulties with recall may also extend to patients’ ratings of experience of care.

We acknowledge that these analyses are probably underpowered with regard to detecting interaction effects, despite the use of a more liberal p value of 0.1 (as opposed to 0.05 for the main effects) in assessing statistical significance. By presenting multiple analyses across the three outcomes, there is also a risk of detecting a statistically significant effect by chance. However, given that the p values for statistically significant main effects of patient characteristics were low (<0.001), we believe our interpretation of effects is largely unaffected.

### Comparison with existing literature

Previous research has identified that certain sociodemographic groups consistently report lower levels of patient satisfaction with primary care services, for example, patients who are young adults or are from ethnic minorities.^2^ ^17^ As reported elsewhere, patients in this trial reported significantly reduced overall satisfaction within the nurse triage arm compared with usual care.^8^ ^9^ We found significantly increased satisfaction among older patients (aged 60–74 years, and 75 years and over), with reduced satisfaction among young adults (16–24 years) and among ethnic minority patients, which is consistent with other research into patient satisfaction, using patient–doctor communication as a proxy for overall satisfaction.^2^ The interactions between trial arm and sociodemographic characteristics such as ethnicity, age and deprivation may be due to differences in factors associated with patient evaluation of care across sociodemographic groups.^3^

Our results are consistent with those of an earlier patient survey within general practice, the GP Access Survey, conducted in 2007–2008.^20^ That survey found that patient employment status was associated with poorer experience of aspects of access to GP care. Among patients in employment, those patients able to take time away from work to visit their GP reported more positive experience, compared with patients unable to do so. More recently, analyses of data from the English GPPS regarding service users' experience of out of hours GP care have indicated poorer experience among service users who were unable to take time away from work to see a GP during their regular working hours, compared with non-working service users.^21^

### Implications for practice and research

Provision of timely care within a primary care setting has many challenges, for example due to patients having difficulty in attending consultations during regular general practice hours. Further research is warranted into how best to serve patients whose employment commitments make attendance at their practice during regular general practice hours difficult or impossible; these analyses provided no indication that a telephone triage system improves the experience of such patients. Lower general satisfaction of ethnic minority patients may be best addressed using qualitative methods to better understand the drivers of satisfaction; our results indicate that rapid access to a GP is of importance and this could be pursued further. Any further research in this field would be enhanced by the inclusion of data regarding the specific nature of the request for urgent care, which would provide the opportunity to explore case type/severity with regard to patient satisfaction.

These additional analyses of patient experience data derived from the ESTEEM trial have enhanced our understanding of patients’ experiences of provision of prompt primary care access, including telephone triage approaches. We have identified patient groups whose reported experience of care following a same-day GP consultation request was less positive than comparator groups, specifically patients from ethnic minorities, school-age children and their parents/guardians, and patients who find it difficult to attend a practice consultation during routine hours. These findings may indicate areas for further research into how best to tailor prompt management in primary care to meet the needs of these patients.

## Supplementary Material

Web appendix
