# Supplementary material for: Exploring demographic and lifestyle associations with patient experience following telephone triage by a primary care doctor or nurse: secondary analyses from a cluster randomised controlled trial
Source: BMJ Qual Saf. 2015 May 18;24(9):572–82. doi: 10.1136/bmjqs-2015-003937 (PMC4552919; doi:10.1136/bmjqs-2015-003937)
Supplement: Web appendix [file bmjqs-2015-003937-s1.pdf]

Online Appendix: Tables A1 to A4

Table A1. Overall satisfaction with care: full results for Model A and non-significant interactions between significant patient characteristics and trial arm (Models H to J)

	Mean difference in overall satisfaction with care[1] (95% CI)	Global *p*-value
**Model A: N=11 343**		
**Trial arm**		
Reference; usual care		
GP triage	1.18 (-0.69 to 3.06)	<0.001
Nurse triage	3.78 (1.88 to 5.69)	
**Patient characteristic**		
Age (reference: 25–59 years)		
Under 5 years	0.90 (-0.62 to 2.42)	
5–11 years	0.60 (-1.21 to 2.41)	
16–24 years	4.35 (2.74 to 5.97)	<0.001
60–74 years	-2.70 (-3.81 to -1.60)	
75 years and over	-3.83 (-5.25 to -2.40)	
Ethnic group (reference; white)		
Other ethnic group	5.00 (2.96 to 7.04)	<0.001
Ease of taking time away from work to attend surgery (reference: not relevant[2])		
Can take time away from work easily	-0.63 (-1.75 to 0.49)	
Can take time away from work with difficulty	2.46 (1.29 to 3.62)	<0.001
Cannot take time away from work	5.41 (3.89 to 6.94)	
Presence of long-standing health condition (reference: none)		
Long-standing health condition present	1.83 (0.98 to 2.67)	<0.001
**Practice characteristic**		
Location (reference: Devon)		
Bristol	0.73 (-1.46 to 2.92)	
Warwick	-0.11 (-2.33 to 2.11)	0.670
Norwich	-0.65 (-2.81 to 1.51)	
Practice size[3] (reference: large)		
Medium	-0.57 (-2.33 to 1.19)	0.068
Small	-3.43 (-6.32 to -0.53)	
Practice deprivation[4] (reference: non-deprived)		
Deprived	1.68 (-0.17 to 3.54)	0.075

**Model H[5]: N=11 343**		
GP triage interaction with age		
Under 5 years	-1.74 (-5.19 to 1.71)	
5–11 years	-0.40 (-4.59 to 3.78)	
16–24 years	0.43 (-3.44 to 4.29)	
60–74 years	-0.29 (-2.58 to 2.00)	
75 years and over	-3.01 (-5.99 to -0.03)	
Nurse triage interaction with age		
Under 5 years	-0.56 (-3.92 to 2.80)	0.302
5–11 years	1.87 (-2.33 to 6.06)	
16–24 years	-2.57 (-6.46 to 1.32)	
60–74 years	-1.15 (-3.51 to 1.21)	
75 years and over	-3.74 (-6.84 to -0.64)	
**Model I[6]: N=11 343**		
GP triage interaction with ease of taking time away from work to attend surgery		
Can take time away from work easily	-0.14 (-2.56 to 2.28)	
Can take time away from work with difficulty	1.18 (-1.17 to 3.53)	
Cannot take time away from work	-1.87 (-5.25 to 1.51)	
Nurse triage interaction with ease of taking time away from work to attend surgery		0.515
Can take time away from work easily	1.23 (-1.24 to 3.70)	
Can take time away from work with difficulty	1.53 (-0.88 to 3.93)	
Cannot take time away from work	-1.53 (-4.82 to 1.76)	
**Model J[7]: N=11 343**		
GP triage interaction with presence of long-standing health condition		
Long-standing health condition present	0.94 (-0.89 to 2.78)	
Nurse triage interaction with presence of long-standing health condition		0.563
Long-standing health condition present	0.77 (-1.10 to 2.63)	

[1]Positive mean difference indicates reduced overall satisfaction with care in comparator group; scale 0–100. [2]Patient does not work, e.g. a child or non-working adult. [3]Small: <3500 patients registered; medium: 3500–8000 patients registered; large: >8000 patients registered. [4]Obtained from Public Health England National General Practice Profiles: http://www.apho.org.uk/PRACPROF/ ; deprived: above average deprivation for England; non-deprived: average/below average deprivation for England. [5]Adjusted as for Model A, with inclusion of interaction between trial arm and age. [6]Adjusted as for Model A, with inclusion of interaction between trial arm and ease of taking time away from work to attend surgery. [7]Adjusted as for Model A, with inclusion of interaction between trial arm and presence of a long-standing health condition. CI: confidence interval. GP: general practitioner.

Table A2. Ease of getting medical help or advice: full results for Model C and non-significant interactions between significant patient characteristics and trial arm (Models K to M)

	Mean difference in ease of getting medical help or advice[1] (95% CI)	Global *p*-value
**Model C: N=10 683**		
**Trial arm**		
Reference; usual care		
GP triage	-0.30 (-2.59; 1.98)	<0.001
Nurse triage	4.81 (2.49; 7.12)	
**Patient characteristic**		
Age (reference: 25–59 years)		
Under 5 years	1.19 (-0.47; 2.86)	
5–11 years	1.54 (-0.44; 3.51)	
16–24 years	2.59 (0.83; 4.35)	<0.001
60–74 years	-3.22 (-4.44; -1.99)	
75 years and over	-3.36 (-4.96; -1.76)	
Ethnic group (reference; white)		
Other ethnic group	5.86 (3.60; 8.12)	<0.001
Ease of taking time away from work to attend surgery (reference: not relevant[2])		
Can take time away from work easily	-0.87 (-2.11; 0.38)	
Can take time away from work with difficulty	3.74 (2.46; 5.03)	<0.001
Cannot take time away from work	7.65 (5.97; 9.32)	
Presence of long-standing health condition (reference: no long-standing health condition)		
Long-standing health condition present	2.01 (1.07; 2.94)	<0.001
**Practice characteristic**		
Location (reference: Devon)		
Bristol	1.14 (-1.52 to 3.81)	
Warwick	1.91 (-0.79 to 4.61)	0.371
Norwich	-0.31 (-2.94 to 2.32)	
Practice size[3] (reference: large)		
Medium	-1.62 (-3.76 to 0.53)	0.002
Small	-6.23 (-9.72 to -2.74)	
Practice deprivation[4] (reference: non-deprived)		
Deprived	3.46 (1.21 to 5.70)	0.003

**Model K[5]: N=10 683**		
GP triage interaction with age		
Under 5 years	-1.51 (-5.26 to 2.25)	
5–11 years	-0.79 (-5.36 to 3.79)	
16–24 years	-0.50 (-4.70 to 3.71)	
60–74 years	0.30 (-2.25 to 2.84)	
75 years and over	-0.98 (-4.35 to 2.40)	
Nurse triage interaction with age		
Under 5 years	-2.10 (-5.77 to 1.57)	0.873
5–11 years	-0.06 (-4.64 to 4.52)	
16–24 years	-3.35 (-7.58 to 0.89)	
60–74 years	0.24 (-2.38 to 2.86)	
75 years and over	-2.01 (-5.52 to 1.50)	
**Model L[6]: N=10 683**		
GP triage interaction with ease of taking time away from work to attend surgery		
Can take time away from work easily	-0.54 (-3.21 to 2.14)	
Can take time away from work with difficulty	-0.54 (-3.13 to 2.05)	
Cannot take time away from work	-1.84 (-5.56 to 1.89)	
Nurse triage interaction with ease of taking time away from work to attend surgery		0.720
Can take time away from work easily	1.41 (-1.32 to 4.15)	
Can take time away from work with difficulty	-0.75 (-3.40 to 1.90)	
Cannot take time away from work	-0.29 (-3.89 to 3.31)	
**Model M[7]: N=10 683**		
GP triage interaction with presence of long-standing health condition		
Long-standing health condition present	-0.09 (-2.12 to 1.94)	
Nurse triage interaction with presence of long-standing health condition		0.546
Long-standing health condition present	0.97 (-1.10 to 3.03)	

[1]Positive mean difference indicates reduced ease of getting medical help or advice in comparator group; scale 0–100. [2]Patient does not work, e.g. a child or non-working adult. [3]Small: <3500 patients registered; medium: 3500–8000 patients registered; large: >8000 patients registered. [4]Obtained from Public Health England National General Practice Profiles: http://www.apho.org.uk/PRACPROF/ ; deprived: above average deprivation for England; non-deprived: average/below average deprivation for England. [5]Adjusted as for Model C, with inclusion of interaction between trial arm and age. [6]Adjusted as for Model C, with inclusion of interaction between trial arm and ease of taking time away from work to attend surgery. [7]Adjusted as for Model C, with inclusion of interaction between trial arm and presence of a long-standing health condition. CI: confidence interval. GP: general practitioner.

Table A3. Convenience of care: socio-demographic interactions with trial arm

	Mean difference in overall satisfaction with care[1] (95% CI)	Global *p*-value	Marginal mean value[2] (95% CI)
**Model F[3]: N=11 243**			
GP triage interaction with age			
Under 5 years	-3.17 (-6.85 to 0.51)		
5–11 years	1.46 (-3.01 to 5.93)		
16–24 years	0.68 (-3.46 to 4.82)		
60–74 years	-0.08 (-2.53 to 2.37)		
75 years and over	-2.69 (-5.90 to 0.51)		
Nurse triage interaction with age		0.072	
Under 5 years	-1.59 (-5.19 to 2.01)		
5–11 years	6.08 (1.59 to 10.57)		
16–24 years	-1.33 (-5.49 to 2.83)		
60–74 years	0.33 (-2.20 to 2.86)		
75 years and over	-3.09 (-6.42 to 0.25)		
Marginal mean values			
Usual care			
Under 5 years			17.7 (14.8 to 20.5)
5–11 years			14.8 (11.5 to 18.2)
16–24 years			17.4 (14.4 to 20.5)
25–59 years			13.0 (11.0 to 14.9)
60–74 years			9.6 (7.4 to 11.7)
75 years and over			12.4 (9.6 to 15.1)
GP triage			
Under 5 years			16.9 (14.0 to 19.9)
5–11 years			18.7 (15.3 to 22.2)
16–24 years			20.6 (17.2 to 23.9)
25–59 years			15.4 (13.5 to 17.4)
60–74 years			11.9 (9.8 to 14.1)
75 years and over			12.1 (9.7 to 14.6)
Nurse triage			
Under 5 years			21.9 (19.1 to 24.7)
5–11 years			26.7 (23.3 to 30.2)
16–24 years			21.9 (18.6 to 25.3)
25–59 years			18.8 (16.9 to 20.8)
60–74 years			15.7 (13.6 to 17.9)
75 years and over			15.1 (12.5 to 17.7)

**Model G[4]: N=11 243**			
GP triage interaction with patient deprivation[5]			
Quintile 2	1.87 (-1.26 to 5.01)		
Quintile 3	1.42 (-1.91 to 4.76)		
Quintile 4	-1.09 (-5.05 to 2.86)		
Quintile 5	-0.18 (-5.84 to 5.48)		
Nurse triage interaction with patient deprivation[5]		0.041	
Quintile 2	-2.90 (-5.92 to 0.13)		
Quintile 3	-3.87 (-7.01 to -0.73)		
Quintile 4	-3.56 (-7.21 to 0.09)		
Quintile 5	-4.96 (-10.28 to 0.35)		
Marginal mean values			
Usual care			
Quintile 1			12.8 (10.4 to 15.1)
Quintile 2			12.0 (9.9 to 14.1)
Quintile 3			12.7 (10.6 to 14.8)
Quintile 4			14.4 (12.1 to 16.7)
Quintile 5			10.6 (6.9 to 14.2)
GP triage			
Quintile 1			14.0 (11.5 to 16.6)
Quintile 2			15.1 (13.0 to 17.3)
Quintile 3			15.4 (13.3 to 17.5)
Quintile 4			14.5 (11.9 to 17.2)
Quintile 5			11.7 (7.8 to 15.5)
Nurse triage			
Quintile 1			21.3 (19.0 to 23.7)
Quintile 2			17.7 (15.5 to 19.9)
Quintile 3			17.4 (15.2 to 19.6)
Quintile 4			19.4 (17.0 to 21.8)
Quintile 5			14.2 (10.7 to 17.7)

[1]Positive mean difference indicates lower convenience of care in comparator group; scale 0–100. [2]Higher value indicates lower convenience of care; scale 0–100. [3]Adjusted as for Model E (Table 4), with inclusion of interaction between trial arm and age. [4]Adjusted as for Model E (Table 4), with inclusion of interaction between trial arm and patient deprivation. [5]IMD 2010 score and rank derived from residential postcode data mapped to lower super output area (LSOA); https://www.gov.uk/government/publications/english-indices-of-deprivation-2010. CI: confidence interval. GP: general practitioner.

Table A4. Convenience of care: full results for Model E and non-significant interactions between significant patient characteristics and trial arm (Models O to Q)

	Mean difference in overall convenience of care[1] (95% CI)	Global *p*-value
**Model E: N=11 243**		
**Trial arm**		
Reference: usual care		
GP triage	1.99 (-0.49; 4.47)	<0.001
Nurse triage	5.69 (3.19; 8.20)	
**Patient characteristic**		
Age (reference: 25–59 years)		
Under 5 years	3.11 (1.48; 4.74)	
5–11 years	4.28 (2.35; 6.21)	
16–24 years	4.24 (2.51; 5.97)	<0.001
60–74 years	-3.35 (-4.54; -2.17)	
75 years and over	-2.68 (-4.21; -1.15)	
Patient deprivation[2] (reference: Quintile 1; least deprived)		
Quintile 2	-1.27 (-2.54; <-0.01)	
Quintile 3	-1.05 (-2.39; -0.28)	0.001
Quintile 4	-0.01 (-1.59; 1.57)	
Quintile 5	-4.05 (-6.35; -1.75)	
Ethnic group (reference: white)		
Other ethnic group	6.36 (4.16; 8.56)	<0.001
Ease of taking time away from work to attend surgery (reference: not relevant[3])		
Can take time away from work easily	-1.56 (-2.76; -0.36)	
Can take time away from work with difficulty	4.64 (3.39; 5.89)	<0.001
Cannot take time away from work	9.02 (7.39; 10.65)	
Presence of long-standing health condition (reference: no long-standing health condition)		
Long-standing health condition present	1.90 (1.00; 2.81)	<0.001
**Practice characteristic**		
Location (reference: Devon)		
Bristol	0.42 (-2.49 to 3.33)	
Warwick	0.87 (-2.07 to 3.81)	0.912
Norwich	-0.16 (-3.01 to 2.68)	
Practice size[4] (reference: large)		
Medium	-1.67 (-3.99 to 0.66)	0.040
Small	-4.54 (-8.28 to -0.80)	
Practice deprivation[5] (reference: non-deprived)		
Deprived	3.51 (1.00 to 6.02)	0.006

**Model O[6]: N=11 243**		
GP triage interaction with ethnicity		
Other ethnic group	-3.14 (-8.44; 2.15)	0.464
Nurse triage interaction with ethnicity		
Other ethnic group	-0.64 (-6.05; 4.76)	
**Model P[7]: N=11 243**		
GP triage interaction with ease of taking time away from work to attend surgery		
Can take time away from work easily	-0.51 (-3.10 to 2.07)	
Can take time away from work with difficulty	0.53 (-1.98 to 3.05)	
Cannot take time away from work	1.66 (-1.95 to 5.26)	
Nurse triage interaction with ease of taking time away from work to attend surgery		0.802
Can take time away from work easily	1.17 (-1.48 to 3.83)	
Can take time away from work with difficulty	1.16 (-1.41 to 3.73)	
Cannot take time away from work	0.78 (-2.75 to 4.30)	
**Model Q[8]: N=11 243**		
GP triage interaction with presence of long-standing health condition		
Long-standing health condition present	0.62 (-1.34 to 2.59)	
Nurse triage interaction with presence of long-standing health condition		0.744
Long-standing health condition present	0.71 (-1.29 to 2.71)	

[1]Positive mean difference indicates lower convenience of care in comparator group; scale 0–100. [2]IMD 2010 score and rank derived from residential postcode data mapped to lower super output area (LSOA); https://www.gov.uk/government/publications/english-indices-of-deprivation-2010. [3]Patient does not work, e.g. a child or non-working adult. [4]Small: <3500 patients registered; medium: 3500–8000 patients registered; large: >8000 patients registered. [5]Obtained from Public Health England National General Practice Profiles: http://www.apho.org.uk/PRACPROF/ ; deprived: above average deprivation for England; non-deprived: average/below average deprivation for England. [6]Adjusted as for Model E, with inclusion of interaction between trial arm and ethnicity. [7]Adjusted as for Model E, with inclusion of interaction between trial arm and ease of taking time away from work to attend surgery. [8]Adjusted as for Model E, with inclusion of interaction between trial arm and presence of a long-standing health condition. CI: confidence interval. GP: general practitioner.
